# Person-centered care assessment tool with a focus on quality healthcare: a systematic review of psychometric properties

**DOI:** 10.1186/s40359-024-01716-7

**Published:** 2024-04-19

**Authors:** Lluna Maria Bru-Luna, Manuel Martí-Vilar, César Merino-Soto, José Livia-Segovia, Juan Garduño-Espinosa, Filiberto Toledano-Toledano

**Affiliations:** 1 Departamento de Educación, Facultad de Ciencias Sociales, Universidad Europea de Valencia, 46010 Valencia, Spain; 2 Departamento de Psicología Básica, Universitat de València, Blasco Ibáñez Avenue, 21, 46010 Valencia, Spain; 3 Departamento de Psicología, Instituto de Investigación de Psicología, Universidad de San Martín de Porres, Tomás Marsano Avenue 242, Lima 34, Perú; 4 Instituto Central de Gestión de la Investigación, Universidad Nacional Federico Villarreal, Carlos Gonzalez Avenue 285, 15088 San Miguel, Perú; 5 Unidad de Investigación en Medicina Basada en Evidencias, Hospital Infantil de México Federico Gómez Instituto Nacional de Salud, Dr. Márquez 162, 06720 Doctores, Cuauhtémoc Mexico; 6 Unidad de Investigación Multidisciplinaria en Salud, Instituto Nacional de Rehabilitación Luis Guillermo Ibarra Ibarra, México-Xochimilco 289, Arenal de Guadalupe, 14389 Tlalpan, Mexico City, Mexico; 7 Dirección de Investigación y Diseminación del Conocimiento, Instituto Nacional de Ciencias e Innovación para la Formación de Comunidad Científica, INDEHUS, Periférico Sur 4860, Arenal de Guadalupe, 14389 Tlalpan, Mexico City, Mexico

**Keywords:** Person-centered care, PCC, Person-centered care assessment tool, P-CAT, Validity, Standards

## Abstract

**Background:**

The person-centered care (PCC) approach plays a fundamental role in ensuring quality healthcare. The Person-Centered Care Assessment Tool (P-CAT) is one of the shortest and simplest tools currently available for measuring PCC. The objective of this study was to conduct a systematic review of the evidence in validation studies of the P-CAT, taking the “Standards” as a frame of reference.

**Methods:**

First, a systematic literature review was conducted following the PRISMA method. Second, a systematic descriptive literature review of validity tests was conducted following the “Standards” framework. The search strategy and information sources were obtained from the Cochrane, Web of Science (WoS), Scopus and PubMed databases. With regard to the eligibility criteria and selection process, a protocol was registered in PROSPERO (CRD42022335866), and articles had to meet criteria for inclusion in the systematic review.

**Results:**

A total of seven articles were included. Empirical evidence indicates that these validations offer a high number of sources related to test content, internal structure for dimensionality and internal consistency. A moderate number of sources pertain to internal structure in terms of test-retest reliability and the relationship with other variables. There is little evidence of response processes, internal structure in measurement invariance terms, and test consequences.

**Discussion:**

The various validations of the P-CAT are not framed in a structured, valid, theory-based procedural framework like the “Standards” are. This can affect clinical practice because people’s health may depend on it. The findings of this study show that validation studies continue to focus on the types of validity traditionally studied and overlook interpretation of the scores in terms of their intended use.

**Supplementary Information:**

The online version contains supplementary material available at 10.1186/s40359-024-01716-7.

## Background

### Person-centered care (PCC)

Quality care for people with chronic diseases, functional limitations, or both has become one of the main objectives of medical and care services. The person-centered care (PCC) approach is an essential element not only in achieving this goal but also in providing high-quality health maintenance and medical care [1–3]. In addition to guaranteeing human rights, PCC provides numerous benefits to both the recipient and the provider [4, 5]. Additionally, PCC includes a set of necessary competencies for healthcare professionals to address ongoing challenges in this area [6]. PCC includes the following elements [7]: an individualized, goal-oriented care plan based on individuals’ preferences; an ongoing review of the plan and the individual’s goals; support from an interprofessional team; active coordination among all medical and care providers and support services; ongoing information exchange, education and training for providers; and quality improvement through feedback from the individual and caregivers.

There is currently a growing body of literature on the application of PCC. A good example of this is McCormack’s widely known mid-range theory [8], an internationally recognized theoretical framework for PCC and how it is operationalized in practice. This framework forms a guide for care practitioners and researchers in hospital settings. This framework is elaborated in PCC and conceived of as “an approach to practice that is established through the formation and fostering of therapeutic relationships between all care providers, service users, and others significant to them, underpinned by values of respect for persons, [the] individual right to self-determination, mutual respect, and understanding” [9].

Thus, as established by PCC, it is important to emphasize that reference to the person who is the focus of care refers not only to the recipient but also to everyone involved in a care interaction [10, 11]. PCC ensures that professionals are trained in relevant skills and methodology since, as discussed above, carers are among the agents who have the greatest impact on the quality of life of the person in need of care [12–14]. Furthermore, due to the high burden of caregiving, it is essential to account for caregivers’ well-being. In this regard, studies on professional caregivers are beginning to suggest that the provision of PCC can produce multiple benefits for both the care recipient and the caregiver [15].

Despite a considerable body of literature and the frequent inclusion of the term in health policy and research [16], PCC involves several complications. There is no standard consensus on the definition of this concept [17], which includes problematic areas such as efficacy assessment [18, 19]. In addition, the difficulty of measuring the subjectivity involved in identifying the dimensions of the CPC and the infrequent use of standardized measures are acute issues [20]. These limitations and purposes motivated the creation of the Person-Centered Care Assessment Tool (P-CAT; [21]), which emerged from the need for a brief, economical, easily applied, versatile and comprehensive assessment instrument to provide valid and reliable measures of PCC for research purposes [21].

### Person-centered care assessment tool (P-CAT)

There are several instruments that can measure PCC from different perspectives (i.e., the caregiver or the care recipient) and in different contexts (e.g., hospitals and nursing homes). However, from a practical point of view, the P-CAT is one of the shortest and simplest tools and contains all the essential elements of PCC described in the literature. It was developed in Australia to measure the approach of long-term residential settings to older people with dementia, although it is increasingly used in other healthcare settings, such as oncology units [22] and psychiatric hospitals [23].

Due to the brevity and simplicity of its application, the versatility of its use in different medical and care contexts, and its potential emic characteristics (i.e., constructs that can be cross-culturally applicable with reasonable and similar structure and interpretation; [24]), the P-CAT is one of the most widely used tests by professionals to measure PCC [25, 26]. It has expanded to several countries with cultural and linguistic differences. Since its creation, it has been adapted in countries separated by wide cultural and linguistic differences, such as Norway [27], Sweden [28], China [29], South Korea [30], Spain [25], and Italy [31].

The P-CAT comprises 13 items rated on a 5-point ordinal scale (from “strongly disagree” to “strongly agree”), with high scores indicating a high degree of person-centeredness. The scale consists of three dimensions: person-centered care (7 items), organizational support (4 items) and environmental accessibility (2 items). In the original study (*n* = 220; [21]), the internal consistency of the instrument yielded satisfactory values for the total scale (*α* = 0.84) and good test-retest reliability (*r* =.66) at one-week intervals. A reliability generalization study conducted in 2021 [32] that estimated the internal consistency of the P-CAT and analyzed possible factors that could affect the it revealed that the mean *α* value for the 25 meta-analysis samples (some of which were part of the validations included in this study) was 0.81, and the only variable that had a statistically significant relationship with the reliability coefficient was the mean age of the sample. With respect to internal structure validity, three factors (56% of the total variance) were obtained, and content validity was assessed by experts, literature reviews and stakeholders [33].

Although not explicitly stated, the apparent commonality between validation studies of different versions of the P-CAT may be influenced by an influential decades-old validity framework that differentiates three categories: content validity, construct validity, and criterion validity [34, 35]. However, a reformulation of the validity of the P-CAT within a modern framework, which would provide a different definition of validity, has not been performed.

### Scale validity

Traditionally, validation is a process focused on the psychometric properties of a measurement instrument [36]. In the early 20th century, with the frequent use of standardized measurement tests in education and psychology, two definitions emerged: the first defined validity as the degree to which a test measures what it intends to measure, while the second described the validity of an instrument in terms of the correlation it presents with a variable [35].

However, in the past century, validity theory has evolved, leading to the understanding that validity should be based on specific interpretations for an intended purpose. It should not be limited to empirically obtained psychometric properties but should also be supported by the theory underlying the construct measured. Thus, to speak of classical or modern validity theory suggests an evolution in the classical or modern understanding of the concept of validity. Therefore, a classical approach (called classical test theory, CTT) is specifically differentiated from a modern approach. In general, recent concepts associated with a modern view of validity are based on (a) a unitary conception of validity and (b) validity judgments based on inferences and interpretations of the scores of a measure [37, 38]. This conceptual advance in the concept of validity led to the creation of a guiding framework to for obtaining evidence to support the use and interpretation of the scores obtained by a measure [39].

This purpose is addressed by the Standards for Educational and Psychological Testing (“Standards”), a guide created by the American Educational Research Association (AERA), the American Psychological Association (APA) and the National Council on Measurement in Education (NCME) in 2014 with the aim of providing guidelines to assess the validity of the interpretations of scores of an instrument based on their intended use. Two conceptual aspects stand out in this modern view of validity: first, validity is a unitary concept centered on the construct; second, validity is defined as “the degree to which evidence and theory support the interpretations of test scores for proposed uses of tests” [37]. Thus, the “Standards” propose several sources that serve as a reference for assessing different aspects of validity. The five sources of valid evidence are as follows [37]: test content, response processes, internal structure, relations to other variables and consequences of testing. According to AERA et al. [37], test content validity refers to the relationship of the administration process, subject matter, wording and format of test items to the construct they are intended to measure. It is measured predominantly with qualitative methods but without excluding quantitative approaches. The validity of the responses is based on analysis of the cognitive processes and interpretation of the items by respondents and is measured with qualitative methods. Internal structure validity is based on the interrelationship between the items and the construct and is measured by quantitative methods. Validity in terms of the relationship with other variables is based on comparison between the variable that the instrument intends to measure and other theoretically relevant external variables and is measured by quantitative methods. Finally, validity based on the results of the test analyses consequences, both intended and unintended, that may be due to a source of invalidity. It is measured mainly by qualitative methods.

Thus, although validity plays a fundamental role in providing a strong scientific basis for interpretations of test scores, validation studies in the health field have traditionally focused on content validity, criterion validity and construct validity and have overlooked the interpretation and use of scores [34].

“Standards” are considered a suitable validity theory-based procedural framework for reviewing the validity of questionnaires due to its ability to analyze sources of validity from both qualitative and quantitative approaches and its evidence-based method [35]. Nevertheless, due to a lack of knowledge or the lack of a systematic description protocol, very few instruments to date have been reviewed within the framework of the “Standards” [39].

### Current study

Although the P-CAT is one of the most widely used instruments by professionals and has seven validations [25, 27–31, 40], no analysis has been conducted of its validity within the framework of the “Standards”. That is, empirical evidence of the validity of the P-CAT has not been obtained in a way that helps to develop a judgment based on a synthesis of the available information.

A review of this type is critical given that some methodological issues seem to have not been resolved in the P-CAT. For example, although the multidimensionality of the P-CAT was identified in the study that introduced it, Bru-Luna et al. [32] recently stated that in adaptations of the P-CAT [25, 27–30, 40], the total score is used for interpretation and multidimensionality is disregarded. Thus, the multidimensionality of the original study was apparently not replicated. Bru-Luna et al. [32] also indicated that the internal structure validity of the P-CAT is usually underreported due to a lack of sufficiently rigorous approaches to establish with certainty how its scores are calculated.

The validity of the P-CAT, specifically its internal structure, appears to be unresolved. Nevertheless, substantive research and professional practice point to this measure as relevant to assessing PCC. This perception is contestable and judgment-based and may not be sufficient to assess the validity of the P-CAT from a cumulative and synthetic angle based on preceding validation studies. An adequate assessment of validity requires a model to conceptualize validity followed by a review of previous studies of the validity of the P-CAT using this model.

Therefore, the main purpose of this study was to conduct a systematic review of the evidence provided by P-CAT validation studies while taking the “Standards” as a framework.

## Methods

The present study comprises two distinct but interconnected procedures. First, a systematic literature review was conducted following the PRISMA method ( [41]; Additional file 1; Additional file 2) with the aim of collecting all validations of the P-CAT that have been developed. Second, a systematic description of the validity evidence for each of the P-CAT validations found in the systematic review was developed following the “Standards” framework [37]. The work of Hawkins et al. [39], the first study to review validity sources according to the guidelines proposed by the “Standards”, was also used as a reference. Both provided conceptual and pragmatic guidance for organizing and classifying validity evidence for the P-CAT.

The procedure conducted in the systematic review is described below, followed by the procedure for examining the validity studies.

### Systematic review

#### Search strategy and information sources

Initially, the Cochrane database was searched with the aim of identifying systematic reviews of the P-CAT. When no such reviews were found, subsequent preliminary searches were performed in the Web of Science (WoS), Scopus and PubMed databases. These databases play a fundamental role in recent scientific literature since they are the main sources of published articles that undergo high-quality content and editorial review processes [42]. The search formula was as follows. The original P-CAT article [21] was located, after which all articles that cited it through 2021 were identified and analyzed. This approach ensured the inclusion of all validations. No articles were excluded on the basis of language to avoid language bias [43]. Moreover, to reduce the effects of publication bias, a complementary search in Google Scholar was also performed to allow the inclusion of “gray” literature [44]. Finally, a manual search was performed through a review of the references of the included articles to identify other articles that met the search criteria but were not present in any of the aforementioned databases.

This process was conducted by one of the authors and corroborated by another using the Covidence tool [45]. A third author was consulted in case of doubt.

#### Eligibility criteria and selection process

The protocol was registered in PROSPERO, and the search was conducted according to these criteria. The identification code is CRD42022335866.

The articles had to meet the following criteria for inclusion in the systematic review: (a) a methodological approach to P-CAT validations, (b) an experimental or quasiexperimental studies, (c) studies with any type of sample, and (d) studies in any language. We discarded studies that met at least one of the following exclusion criteria: (a) systematic reviews or bibliometric reviews of the instrument or meta-analyses or (b) studies published after 2021.

This process was conducted by one of the authors and corroborated by another using the Covidence tool [45]. A third author was consulted in case of doubt.

#### Data collection process

After the articles were selected, the most relevant information was extracted from each article. Fundamental data were recorded in an Excel spreadsheet for each of the sections: introduction, methodology, results and discussion. Information was also recorded about the limitations mentioned in each article as well as the practical implications and suggestions for future research.

Given the aim of the study, information was collected about the sources of validity of each study, including test content (judges’ evaluation, literature review and translation), response processes, internal structure (factor analysis, design, estimator, factor extraction method, factors and items, interfactor R, internal replication, effect of the method, and factor loadings), and relationships with other variables (convergent, divergent, concurrent and predictive validity) and consequences of measurement.

### Description of the validity study

To assess the validity of the studies, an Excel table was used. Information was recorded for the seven articles included in the systematic review. The data were extracted directly from the texts of the articles and included information about the authors, the year of publication, the country where each P-CAT validation was produced and each of the five standards proposed in the “Standards” [37].

The validity source related to internal structure was divided into three sections to record information about dimensionality (e.g., factor analysis, design, estimator, factor extraction method, factors and items, interfactor R, internal replication, effect of the method, and factor loadings), reliability expression (i.e., internal consistency and test-retest) and the study of factorial invariance according to the groups into which it was divided (e.g., sex, age, profession) and the level of study (i.e., metric, intercepts). This approach allowed much more information to be obtained than relying solely on source validity based on internal structure. This division was performed by the same researcher who performed the previous processes.

## Results

### Systematic review

#### Study selection and study characteristics

The systematic review process was developed according to the PRISMA methodology [41].

The WoS, Scopus, PubMed and Google Scholar databases were searched on February 12, 2022 and yielded a total of 485 articles. Of these, 111 were found in WoS, 114 in Scopus, 43 in PubMed and 217 in Google Scholar. In the first phase, the title and abstracts of all the articles were read. In this first screening, 457 articles were eliminated because they did not include studies with a methodological approach to P-CAT validation and one article was excluded because it was the original P-CAT article. This resulted in a total of 27 articles, 19 of which were duplicated in different databases and, in the case of Google Scholar, within the same database. This process yielded a total of eight articles that were evaluated for eligibility by a complete reading of the text. In this step, one of the articles was excluded due to a lack of access to the full text of the study [31] (although the original manuscript was found, it was impossible to access the complete content; in addition, the authors of the manuscript were contacted, but no reply was received). Finally, a manual search was performed by reviewing the references of the seven studies, but none were considered suitable for inclusion. Thus, the review was conducted with a total of seven articles.

Of the seven studies, six were original validations in other languages. These included Norwegian [27], Swedish [28], Chinese (which has two validations [29, 40]), Spanish [25], and Korean [30]. The study by Selan et al. [46] included a modification of the Swedish version of the P-CAT and explored the psychometric properties of both versions (i.e., the original Swedish version and the modified version).

**Fig. 1:**
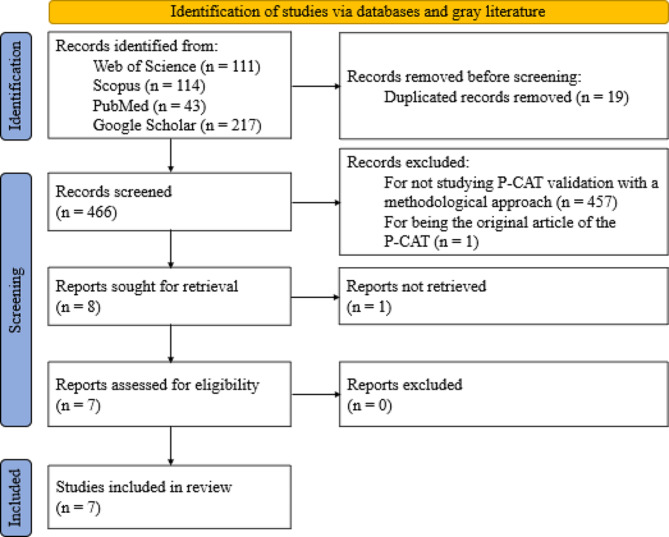
PRISMA 2020 flow diagram for new systematic reviews including database searches

The item selection and screening process are illustrated in detail in Fig. 1.

### Validity analysis

To provide a clear overview of the validity analyses, Table 1 descriptively shows the percentages of items that provide information about the five standards proposed by the “Standards” guide [37].

**Table 1:** Number of studies and percentages for each validity test

Validity evidence	N	%	Validity model
Content	7	100	Classical
Response process	0	0	Classical
Internal structure	-	-	Classical
Dimensionality	7	100	-
Reliability	-	-	-
Internal consistency	7	100	-
Test-retest	4	57	-
Invariance	0	0	-
Relation to other variables	4	57	Classical
Consequences of testing	0	0	Classical

*Note* Validity model: validity theory used (classical, modern)

The table shows a high number of validity sources related to test content and internal structure in relation to dimensionality and internal consistency, followed by a moderate number of sources for test-retest and relationship with other variables. A rate of 0% is observed for validity sources related to response processes, invariance and test consequences. Below, different sections related to each of the standards are shown, and the information is presented in more detail.

#### Evidence based on test content

The first standard, which focused on test content, was met for all items (100%). Translation, which refers to the equivalence of content between the original language and the target language, was met in the six articles that conducted validation in another language and/or culture. These studies reported that the validations were translated by bilingual experts and/or experts in the area of care. In addition, three studies [25, 29, 40] reported that the translation process followed International Test Commission guidelines, such as those of Beaton et al. [47], Guillemin [48], Hambleton et al. [49], and Muñiz et al. [50]. Evaluation by judges, who referred to the relevance, clarity and importance of the content, was divided into two categories: expert evaluation (a panel of expert judges for each of the areas to consider in the evaluation instrument) and experiential evaluation (potential participants testing the test). The first type of evaluation occurred in three of the articles [28, 29, 46], while the other occurred in two [25, 40]. Only one of the items [29] reported that the scale contained items that reflected the dimension described in the literature. The validity evidence related to the test content presented in each article can be found in Table 2.

**Table 2:** Validity tests based on test content

Study	Judges’ assessment		Literature review	Translation
	Experts	Experiential		
Rokstad et al. [27]				X
Sjögren et al. [28]	X			X
Zhong and Lou [29]	X		X	X
Martínez et al. [25]		X		X
Tak et al. [30]				X
Selan et al. [46]	X			
Le et al. [40]		X		X

#### Evidence based on response processes

The second standard, related to the validity of the response process, was obtained according to the “Standards” from the analysis of individual responses: “questioning test takers about their performance strategies or response to particular items (…), maintaining records that monitor the development of a response to a writing task (…), documentation of other aspects of performance, like eye movement or response times…” [37] (p. 15). According to the analysis of the validity of the response processes, none of the articles complied with this evidence.

#### Evidence based on internal structure

The third standard, validity related to internal structure, was divided into three sections. First, the dimensionality of each study was examined in terms of factor analysis, design, estimator, factor extraction method, factors and items, interfactor R, internal replication, effect of the method, and factor loadings. Le et al. [40] conducted an exploratory-confirmatory design while Sjögren et al. [28] conducted a confirmatory-exploratory design to assess construct validity using confirmatory factor analysis (CFA) and investigated it further using exploratory factor analysis (EFA). The remaining articles employed only a single form of factor analysis: three employed EFA, and two employed CFA. Regarding the next point, only three of the articles reported the factor extraction method used, including Kaiser’s eigenvalue, criterion, scree plot test, parallel analysis and Velicer’s MAP test. Instrument validations yielded a total of two factors in five of the seven articles, while one yielded a single dimension [25] and the other yielded three dimensions [29], as in the original instrument. The interfactor R was reported only in the study by Zhong and Lou [29], whereas in the study by Martínez et al. [25], it could be easily obtained since it consisted of only one dimension. Internal replication was also calculated in the Spanish validation by randomly splitting the sample into two to test the correlations between factors. The effectiveness of the method was not reported in any of the articles. This information is presented in Table 3 in addition to a summary of the factor loadings.

**Table 3:** Validity tests based on internal structure: dimensionality

Study	Factor analysis	Design	Estimator	Factor extraction method	Factors and items	Interfactor R	Internal replication	Effect of the method	Factor loadings (summary)		
Rokstad et al. [27]	EFA	Expl.	Expl.: PCA, varimax, direct oblimin rotation	Kaiser’s eigenvalue, criterion and scree plot test	F1 = 1, 2, 3, 4, 5, 6, 7, 13 F2 = 8, 9, 10, 11, 12	N.R.	N.R.	N.R.	F1 Max.: 0.738 Min.: 0.515 Average: 0.608	F2 Max.: 0.714 Min.: 0.543 Average: 0.664	
Sjögren et al. [28]	CFA and EFA	Conf. ◊ Expl.	Conf.: ML Expl.: PCA, direct oblimin rotation, orthogonal rotation	Parallel analysis and Velicer’s MAP test	F1 = 1, 2, 3, 4, 5, 6, 11, 13 F2 = 7, 8, 9, 10 12	N.R.	N.R.	N.R.	F1 Max.: 0.7 Min.: 0.4 Average: 0.593	F2 Max.: 0.75 Min.: 0.46 Average: 0.672	
Zhong and Lou [29]	CFA	Conf.	Conf: N.R.	N.R.	F1 = 1, 2, 3, 4, 5, 6 F2 = 7, 8, 9, 10, 11, 12 F3 = 13, 14, 15	P-CAT-C1– P-CAT-C2: 0.043 P-CAT-C1– P-CAT-C3: 0.23 P-CAT-C2– P-CAT-C3: 0.065	N.R.	N.R.	F1 Max.: 0.666 Min.: 0.443 Average: 0.51	F2 Max.: 0.729 Min.: 0.454 Average: 0.584	F3 Max.: 0.51 Min.: 0.399 Average: 0.455
Martínez et al. [25]	CFA	Conf.	Conf.: WLSMV	N.R.	F1 = 1, 2, 3, 4, 5, 6, 7, 8, 9, 10, 11, 12, 13	1	The total sample was divided into two random subsamples. In the two-dimensional model, the correlation between the two factors was analyzed in both subsamples.	N.R.	F1 Max.: 0.73 Min.: 0.33 Average: 0.56		
Tak et al. [30]	EFA	Expl.	Expl.: PCA, varimax orthogonal rotation	N.R.	F1 = 1, 2, 3, 4, 5, 6, 7 F2 = 8, 9, 10, 11, 12, 13	N.R.	N.R.	N.R.	F1 Max.: 0.78 Min.: 0.5 Average: 0.692	F2 Max.: 0.8 Min.: 0.34 Average: 0.653	
Selan et al. [46]	EFA	Expl.	Expl.: PCA, varimax orthogonal rotation	Kaiser’s eigenvalue, criterion, scree plot test and parallel analysis	F1 = 1, 2, 3, 4, 5, 6, 11, 13 F2 = 7, 8, 9, 10, 12	N.R.	N.R.	N.R.	F1 Max.: 0.776 Min.: 0.465 Average: 0.599	F2 Max.: 0.771 Min.: 0.546 Average: 0.711	
Le et al. [40]	EFA and CFA	Expl. ◊ Conf.	Expl.: PCA, varimax rotation, oblique rotation Conf.: SEM	N.R.	F1 = 1, 2, 3, 4, 5, 6, 7, 10, 13 F2 = 8, 9, 11, 12	N.R.	N.R.	N.R.	F1 Max.: 0.79 Min.: 0.17 Average: 0.596	F2 Max.: 0.89 Min.: 0.71 Average: 0.817	

*Note* N.R.: not reported. CFA: confirmatory factor analysis. EFA: exploratory factor analysis. Expl.: exploratory. Conf.: confirmatory. PCA: principal component analysis. MAP test: minimum average partial test. WLSMV: weighted least squares means and variance adjusted estimator. SEM: structural equation modeling. Max.: maximum factor loading. Min: minimum factor loading. Average: average factor loading

The second section examined reliability. All the studies presented measures of internal consistency conducted in their entirety with Cronbach’s *α* coefficient for both the total scale and the subscales. The *ω* coefficient of McDonald was not used in any case. Four of the seven articles performed a test-retest test. Martínez et al. [25] conducted a test-retest after a period of seven days, while Le et al. [40] and Rokstad et al. [27] performed it between one and two weeks later and Sjögren et al. [28] allowed approximately two weeks to pass after the initial test.

The third section analyzes the calculation of invariance, which was not reported in any of the studies.

#### Evidence based on relationships with other variables

In the fourth standard, based on validity according to the relationship with other variables, the articles that reported it used only convergent validity (i.e., it was hypothesized that the variables related to the construct measured by the test—in this case, person-centeredness—were positively or negatively related to another construct). Discriminant validity hypothesizes that the variables related to the PCC construct are not correlated in any way with any other variable studied. No article (0%) measured discriminant evidence, while four (57%) measured convergent evidence [25, 29, 30, 46]. Convergent validity was obtained through comparisons with instruments such as the Person-Centered Climate Questionnaire–Staff Version (PCQ-S), the Staff-Based Measures of Individualized Care for Institutionalized Persons with Dementia (IC), the Caregiver Psychological Elder Abuse Behavior Scale (CPEAB), the Organizational Climate (CLIOR) and the Maslach Burnout Inventory (MBI). In the case of Selan et al. [46], convergent validity was assessed on two items considered by the authors as “crude measures of person-centered care (i.e., external constructs) giving an indication of the instruments’ ability to measure PCC” (p. 4). Concurrent validity, which measures the degree to which the results of one test are or are not similar to those of another test conducted at more or less the same time with the same participants, and predictive validity, which allows predictions to be established regarding behavior based on comparison between the values of the instrument and the criterion, were not reported in any of the studies.

#### Evidence based on the consequences of testing

The fifth and final standard was related to the consequences of the test. It analyzed the consequences, both intended and unintended, of applying the test to a given sample. None of the articles presented explicit or implicit evidence of this.

The last two sources of validity can be seen in Table 4.

**Table 4:** Evidence of validity based on associations with other variables and the consequences of testing

Study	Relation to other variables	Consequences of testing
Rokstad et al. [27]	Convergent: N.R. Divergent: N.R. Concurrent: N.R. Predictive: N.R.	N.R.
Sjögren et al. [28]	Convergent: N.R. Divergent: N.R. Concurrent: N.R. Predictive: N.R.	N.R.
Zhong and Lou [29]	Convergent: IC and CPEAB. Divergent: N.R. Concurrent: N.R. Predictive: N.R.	N.R.
Martínez et al. [25]	Convergent: emotional exhaustion, depersonalization, personal achievement, and organizational climate. Divergent: N.R. Concurrent: N.R. Predictive: N.R.	N.R.
Tak et al. [30]	Convergent: PCQ-S. Divergent: N.R. Concurrent: N.R. Predictive: N.R.	N.R.
Selan et al. [46]	Convergent: control questions: “The care here is person-centered” and “We work from the individual’s self-perceived needs”. Divergent: N.R. Concurrent: N.R. Predictive: N.R.	N.R.
Le et al. [40]	Convergent: N.R. Divergent: N.R. Concurrent: N.R. Predictive: N.R.	N.R.

*Note* N.R.: not reported. IC: Staff-Based Measures of Individualized Care for Institutionalized Persons with Dementia. CPEAB: Caregiver Psychological Elder Abuse Behavior Scale. PCQ-S: Person-Centered Climate Questionnaire-Staff Version

Table 5 shows the results of the set of validity tests for each study according to the described standards.

**Table 5:** Results of validity tests

Study	Test content	Response processes	Internal structure				Relation to other variables	Consequences of testing
			Factor analysis	Reliability		Invariance		
				Internal consistency	Test-retest			
Rokstad et al. [27]	Processes of translation and cultural adaptation	N.R.	EFA	Cronbach’s alpha	1–2 weeks.	N.R.	N.R.	N.R.
Sjögren et al. [28]	Evaluation by expert judges and processes of translation and cultural adaptation	N.R.	CFA and EFA	Cronbach’s alpha	2 weeks.	N.R.	N.R.	N.R.
Zhong and Lou [29]	Evaluation by expert judges, processes of translation and cultural adaptation, and literature review	N.R.	CFA.	Cronbach’s alpha	N.R.	N.R.	Convergent validity: IC and CPEAB	N.R.
Martínez et al. [25]	Experiential evaluation by judges and processes of translation and cultural adaptation	N.R.	CFA	Cronbach’s alpha	1 week.	N.R.	Convergent validity: emotional exhaustion, depersonalization, personal achievement and organizational climate.	N.R.
Tak et al. [30]	Processes of translation and cultural adaptation	N.R.	EFA	Cronbach’s alpha	N.R.	N.R.	Convergent validity: PCQ-S	N.R.
Selan et al. [46]	Evaluation by expert judges	N.R.	EFA	Cronbach’s alpha	N.R.	N.R. .	Convergent validity: examined by calculating Spearman’s rho correlation between the factors and the control questions	N.R.
Le et al. [40]	Experiential evaluation by judges and processes of translation and cultural adaptation	N.R.	EFA and CFA	Cronbach’s alpha	1–2 weeks.	N.R.	N.R.	N.R.

*Note* N.R.: not reported. CFA: confirmatory factor analysis. EFA: exploratory factor analysis. IC: Staff-Based Measures of Individualized Care for Institutionalized Persons with Dementia. CPEAB: Caregiver Psychological Elder Abuse Behavior Scale. PCQ-S: Person-Centered Climate Questionnaire-Staff Version

## Discussion

The main purpose of this article is to analyze the evidence of validity in different validation studies of the P-CAT. To gather all existing validations, a systematic review of all literature citing this instrument was conducted.

The publication of validation studies of the P-CAT has been constant over the years. Since the publication of the original instrument in 2010, seven validations have been published in other languages (taking into account the Italian version by Brugnolli et al. [31], which could not be included in this study) as well as a modification of one of these versions. The very unequal distribution of validations between languages and countries is striking. A recent systematic review [51] revealed that in Europe, the countries where the PCC approach is most widely used are the United Kingdom, Sweden, the Netherlands, Northern Ireland, and Norway. It has also been shown that the neighboring countries seem to exert an influence on each other due to proximity [52] such that they tend to organize healthcare in a similar way, as is the case for Scandinavian countries. This favors the expansion of PCC and explains the numerous validations we found in this geographical area.

Although this approach is conceived as an essential element of healthcare for most governments [53], PCC varies according to the different definitions and interpretations attributed to it, which can cause confusion in its application (e.g., between Norway and the United Kingdom [54]). Moreover, facilitators of or barriers to implementation depend on the context and level of development of each country, and financial support remains one of the main factors in this regard [53]. This fact explains why PCC is not globally widespread among all territories. In countries where access to healthcare for all remains out of reach for economic reasons, the application of this approach takes a back seat, as does the validation of its assessment tools. In contrast, in a large part of Europe or in countries such as China or South Korea that have experienced decades of rapid economic development, patients are willing to be involved in their medical treatment and enjoy more satisfying and efficient medical experiences and environments [55], which facilitates the expansion of validations of instruments such as the P-CAT.

Regarding validity testing, the guidelines proposed by the “Standards” [37] were followed. According to the analysis of the different validations of the P-CAT instrument, none of the studies used a structured validity theory-based procedural framework for conducting validation. The most frequently reported validity tests were on the content of the test and two of the sections into which the internal structure was divided (i.e., dimensionality and internal consistency).

In the present article, the most cited source of validity in the studies was the content of the test because most of the articles were validations of the P-CAT in other languages, and the authors reported that the translation procedure was conducted by experts in all cases. In addition, several of the studies employed International Test Commission guidelines, such as those by Beaton et al. [47], Guillemin [48], Hambleton et al. [49], and Muñiz et al. [50]. Several studies also assessed the relevance, clarity and importance of the content.

The third source of validity, internal structure, was the next most often reported, although it appeared unevenly among the three sections into which this evidence was divided. Dimensionality and internal consistency were reported in all studies, followed by test-retest consistency. In relation to the first section, factor analysis, a total of five EFAs and four CFAs were presented in the validations. Traditionally, EFA has been used in research to assess dimensionality and identify key psychological constructs, although this approach involves a number of inconveniences, such as difficulty testing measurement invariance and incorporating latent factors into subsequent analyses [56] or the major problem of factor loading matrix rotation [57]. Studies eventually began to employ CFA, a technique that overcame some of these obstacles [56] but had other drawbacks; for example, the strict requirement of zero cross-loadings often does not fit the data well, and misspecification of zero loadings tends to produce distorted factors [57]. Recently, exploratory structural equation modeling (ESEM) has been proposed. This technique is widely recommended both conceptually and empirically to assess the internal structure of psychological tools [58] since it overcomes the limitations of EFA and CFA in estimating their parameters [56, 57].

The next section, reliability, reports the total number of items according to Cronbach’s *α* reliability coefficient. Reliability is defined as a combination of systematic and random influences that determine the observed scores on a psychological test. Reporting the reliability measure ensures that item-based scores are consistent, that the tool’s responses are replicable and that they are not modified solely by random noise [59, 60]. Currently, the most commonly employed reliability coefficient in studies with a multi-item measurement scale (MIMS) is Cronbach’s *α* [60, 61].

Cronbach’s *α* [62] is based on numerous strict assumptions (e.g., the test must be unidimensional, factor loadings must be equal for all items and item errors should not covary) to estimate internal consistency. These assumptions are difficult to meet, and their violation may produce small reliability estimates [60]. One of the alternative measures to *α* that is increasingly recommended by the scientific literature is McDonald’s *ω* [63], a composite reliability measure. This coefficient is recommended for congeneric scales in which tau equivalence is not assumed. It has several advantages. For example, estimates of *ω* are usually robust when the estimated model contains more factors than the true model, even with small samples, or when skewness in univariate item distributions produces lower biases than those found when using *α* [59].

The test-retest method was the next most commonly reported internal structure section in these studies. This type of reliability considers the consistency of the scores of a test between two measurements separated by a period [64]. It is striking that test-retest consistency does not have a prevalence similar to that of internal consistency since, unlike internal consistency, test-retest consistency can be assessed for practically all types of patient-reported outcomes. It is even considered by some measurement experts to report reliability with greater relevance than internal consistency since it plays a fundamental role in the calculation of parameters for health measures [64]. However, the literature provides little guidance regarding the assessment of this type of reliability.

The internal structure section that was least frequently reported in the studies in this review was invariance. A lack of invariance refers to a difference between scores on a test that is not explained by group differences in the structure it is intended to measure [65]. The invariance of the measure should be emphasized as a prerequisite in comparisons between groups since “if scale invariance is not examined, item bias may not be fully recognized and this may lead to a distorted interpretation of the bias in a particular psychological measure” [65].

Evidence related to other variables was the next most reported source of validity in the studies included in this review. Specifically, the four studies that reported this evidence did so according to convergent validity and cited several instruments. None of the studies included evidence of discriminant validity, although this may be because there are currently several obstacles related to the measurement of this type of validity [66]. On the one hand, different definitions are used in the applied literature, which makes its evaluation difficult; on the other hand, the literature on discriminant validity focuses on techniques that require the use of multiple measurement methods, which often seem to have been introduced without sufficient evidence or are applied randomly.

Validity related to response processes was not reported by any of the studies. There are several methods to analyze this validity. These methods can be divided into two groups: “those that directly access the psychological processes or cognitive operations (think aloud, focus group, and interviews), compared to those which provide indirect indicators which in turn require additional inference (eye tracking and response times)” [38]. However, this validity evidence has traditionally been reported less frequently than others in most studies, perhaps because there are fewer clear and accepted practices on how to design or report these studies [67].

Finally, the consequences of testing were not reported in any of the studies. There is debate regarding this source of validity, with two main opposing streams of thought. On the one hand [68, 69]) suggests that consequences that appear after the application of a test should not derive from any source of test invalidity and that “adverse consequences only undermine the validity of an assessment if they can be attributed to a problem of fit between the test and the construct” (p. 6). In contrast, Cronbach [69, 70] notes that adverse social consequences that may result from the application of a test may call into question the validity of the test. However, the potential risks that may arise from the application of a test should be minimized in any case, especially in regard to health assessments. To this end, it is essential that this aspect be assessed by instrument developers and that the experiences of respondents be protected through the development of comprehensive and informed practices [39].

This work is not without limitations. First, not all published validation studies of the P-CAT, such as the Italian version by Brugnolli et al. [31], were available. These studies could have provided relevant information. Second, many sources of validity could not be analyzed because the studies provided scant or no data, such as response processes [25, 27–30, 40, 46], relationships with other variables [27, 28, 40], consequences of testing [25, 27–30, 40, 46], or invariance [25, 27–30, 40, 46] in the case of internal structure and interfactor R [27, 28, 30, 40, 46], internal replication [27–30, 40, 46] or the effect of the method [25, 27–30, 40, 46] in the case of dimensionality. In the future, it is hoped that authors will become aware of the importance of validity, as shown in this article and many others, and provide data on unreported sources so that comprehensive validity studies can be performed.

The present work also has several strengths. The search was extensive, and many studies were obtained using three different databases, including WoS, one of the most widely used and authoritative databases in the world. This database includes a large number and variety of articles and is not fully automated due to its human team [71–73]. In addition, to prevent publication bias, gray literature search engines such as Google Scholar were used to avoid the exclusion of unpublished research [44]. Finally, linguistic bias was prevented by not limiting the search to articles published in only one or two languages, thus avoiding the overrepresentation of studies in one language and underrepresentation in others [43].

## Conclusions

Validity is understood as the degree to which tests and theory support the interpretations of instrument scores for their intended use [37]. From this perspective, the various validations of the P-CAT are not presented in a structured, valid, theory-based procedural framework like the “Standards” are. After integration and analysis of the results, it was observed that these validation reports offer a high number of sources of validity related to test content, internal structure in dimensionality and internal consistency, a moderate number of sources for internal structure in terms of test-retest reliability and the relationship with other variables, and a very low number of sources for response processes, internal structure in terms of invariance, and test consequences.

Validity plays a fundamental role in ensuring a sound scientific basis for test interpretations because it provides evidence of the extent to which the data provided by the test are valid for the intended purpose. This can affect clinical practice as people’s health may depend on it. In this sense, the “Standards” are considered a suitable and valid theory-based procedural framework for studying this modern conception of questionnaire validity, which should be taken into account in future research in this area.

Although the P-CAT is one of the most widely used instruments for assessing PCC, as shown in this study, PCC has rarely been studied. The developers of measurement tests applied to the health care setting, on which the health and quality of life of many people may depend, should use this validity framework to reflect the clear purpose of the measurement. This approach is important because the equity of decision making by healthcare professionals in daily clinical practice may depend on the source of validity. Through a more extensive study of validity that includes the interpretation of scores in terms of their intended use, the applicability of the P-CAT, an instrument that was initially developed for long-term care homes for elderly people, could be expanded to other care settings. However, the findings of this study show that validation studies continue to focus on traditionally studied types of validity and overlook the interpretation of scores in terms of their intended use.

### Electronic supplementary material

Below is the link to the electronic supplementary material.

Supplementary Material 1

## Data Availability

All data relevant to the study were included in the article or uploaded as additional files. Additional template data extraction forms are available from the corresponding author upon reasonable request.

## Abbreviations

AERA: American Educational Research Association
APA: American Psychological Association
CFA: Confirmatory factor analysis
CLIOR: Organizational Climate
CPEAB: Caregiver Psychological Elder Abuse Behavior Scale
EFA: Exploratory factor analysis
ESEM: Exploratory structural equation modeling
IC: Staff-based Measures of Individualized Care for Institutionalized Persons with Dementia
MBI: Maslach Burnout Inventory
MIMS: Multi-item measurement scale
ML: Maximum likelihood
NCME: National Council on Measurement in Education
P-CAT: Person-Centered Care Assessment Tool
PCC: Person-centered care
PCQ-S: Person-Centered Climate Questionnaire–Staff Version
PRISMA: Preferred Reporting Items for Systematic Reviews and Meta-Analyses
PROSPERO: International Register of Systematic Review Protocols
Standards: Standards for Educational and Psychological Testing
WLSMV: weighted least square mean and variance adjusted
WoS: Web of Science

## References

[1] Institute of Medicine (2001). Crossing the quality chasm: a new health system for the 21st century.

[2] International Alliance of Patients’ Organizations (2007). What is patient-centred healthcare? A review of definitions and principles.

[3] World Health Organization (2015). WHO global strategy on people-centred and integrated health services: interim report.

[4] Britten N, Ekman I, Naldemirci Ö, Javinger M, Hedman H, Wolf A (2020). Learning from Gothenburg model of person centred healthcare. BMJ.

[5] Van Diepen C, Fors A, Ekman I, Hensing G (2020). Association between person-centred care and healthcare providers’ job satisfaction and work-related health: a scoping review. BMJ Open.

[6] Ekman N, Taft C, Moons P, Mäkitalo Å, Boström E, Fors A (2020). A state-of-the-art review of direct observation tools for assessing competency in person-centred care. Int J Nurs Stud.

[7] American Geriatrics Society Expert Panel on Person-Centered Care (2016). Person-centered care: a definition and essential elements. J Am Geriatr Soc.

[8] McCormack B, McCance TV (2006). Development of a framework for person-centred nursing. J Adv Nurs.

[9] McCormack B, McCance T (2016). Person-centred practice in nursing and health care: theory and practice.

[10] Nolan MR, Davies S, Brown J, Keady J, Nolan J (2004). Beyond person-centred care: a new vision for gerontological nursing. J Clin Nurs.

[11] McCormack B, McCance T (2010). Person-centred nursing: theory, models and methods.

[12] Abraha I, Rimland JM, Trotta FM, Dell’Aquila G, Cruz-Jentoft A, Petrovic M (2017). Systematic review of systematic reviews of non-pharmacological interventions to treat behavioural disturbances in older patients with dementia. The SENATOR-OnTop series. BMJ Open.

[13] Anderson K, Blair A (2020). Why we need to care about the care: a longitudinal study linking the quality of residential dementia care to residents’ quality of life. Arch Gerontol Geriatr.

[14] Bauer M, Fetherstonhaugh D, Haesler E, Beattie E, Hill KD, Poulos CJ (2018). The impact of nurse and care staff education on the functional ability and quality of life of people living with dementia in aged care: a systematic review. Nurse Educ Today.

[15] Smythe A, Jenkins C, Galant-Miecznikowska M, Dyer J, Downs M, Bentham P (2020). A qualitative study exploring nursing home nurses’ experiences of training in person centred dementia care on burnout. Nurse Educ Pract.

[16] McCormack B, Borg M, Cardiff S, Dewing J, Jacobs G, Janes N (2015). Person-centredness– the ‘state’ of the art. Int Pract Dev J.

[17] Wilberforce M, Challis D, Davies L, Kelly MP, Roberts C, Loynes N (2016). Person-centredness in the care of older adults: a systematic review of questionnaire-based scales and their measurement properties. BMC Geriatr.

[18] Rathert C, Wyrwich MD, Boren SA (2013). Patient-centered care and outcomes: a systematic review of the literature. Med Care Res Rev.

[19] Sharma T, Bamford M, Dodman D (2016). Person-centred care: an overview of reviews. Contemp Nurse.

[20] Ahmed S, Djurkovic A, Manalili K, Sahota B, Santana MJ (2019). A qualitative study on measuring patient-centered care: perspectives from clinician-scientists and quality improvement experts. Health Sci Rep.

[21] Edvardsson D, Fetherstonhaugh D, Nay R, Gibson S (2010). Development and initial testing of the person-centered Care Assessment Tool (P-CAT). Int Psychogeriatr.

[22] Tamagawa R, Groff S, Anderson J, Champ S, Deiure A, Looyis J (2016). Effects of a provincial-wide implementation of screening for distress on healthcare professionals’ confidence and understanding of person-centered care in oncology. J Natl Compr Canc Netw.

[23] Degl’ Innocenti A, Wijk H, Kullgren A, Alexiou E (2020). The influence of evidence-based design on staff perceptions of a supportive environment for person-centered care in forensic psychiatry. J Forensic Nurs.

[24] Hulin CL (1987). A psychometric theory of evaluations of item and scale translations: fidelity across languages. J Cross Cult Psychol.

[25] Martínez T, Suárez-Álvarez J, Yanguas J, Muñiz J (2016). Spanish validation of the person-centered Care Assessment Tool (P-CAT). Aging Ment Health.

[26] Martínez T, Martínez-Loredo V, Cuesta M, Muñiz J (2020). Assessment of person-centered care in gerontology services: a new tool for healthcare professionals. Int J Clin Health Psychol.

[27] Rokstad AM, Engedal K, Edvardsson D, Selbaek G (2012). Psychometric evaluation of the Norwegian version of the person-centred Care Assessment Tool. Int J Nurs Pract.

[28] Sjögren K, Lindkvist M, Sandman PO, Zingmark K, Edvardsson D (2012). Psychometric evaluation of the Swedish version of the person-centered Care Assessment Tool (P-CAT). Int Psychogeriatr.

[29] Zhong XB, Lou VW (2013). Person-centered care in Chinese residential care facilities: a preliminary measure. Aging Ment Health.

[30] Tak YR, Woo HY, You SY, Kim JH (2015). Validity and reliability of the person-centered Care Assessment Tool in long-term care facilities in Korea. J Korean Acad Nurs.

[31] Brugnolli A, Debiasi M, Zenere A, Zanolin ME, Baggia M (2020). The person-centered Care Assessment Tool in nursing homes: psychometric evaluation of the Italian version. J Nurs Meas.

[32] Bru-Luna LM, Martí-Vilar M, Merino-Soto C, Livia J (2021). Reliability generalization study of the person-centered Care Assessment Tool. Front Psychol.

[33] Edvardsson D, Innes A (2010). Measuring person-centered care: a critical comparative review of published tools. Gerontologist.

[34] Hawkins M, Elsworth GR, Nolte S, Osborne RH (2021). Validity arguments for patient-reported outcomes: justifying the intended interpretation and use of data. J Patient Rep Outcomes.

[35] Sireci SG (2016). On the validity of useless tests. Assess Educ Princ Policy Pract.

[36] Hawkins M, Elsworth GR, Osborne RH (2019). Questionnaire validation practice: a protocol for a systematic descriptive literature review of health literacy assessments. BMJ Open.

[37] American Educational Research Association, American Psychological Association (2014). National Council on Measurement in Education. Standards for educational and psychological testing.

[38] Padilla JL, Benítez I (2014). Validity evidence based on response processes. Psicothema.

[39] Hawkins M, Elsworth GR, Hoban E, Osborne RH (2020). Questionnaire validation practice within a theoretical framework: a systematic descriptive literature review of health literacy assessments. BMJ Open.

[40] Le C, Ma K, Tang P, Edvardsson D, Behm L, Zhang J (2020). Psychometric evaluation of the Chinese version of the person-centred Care Assessment Tool. BMJ Open.

[41] Page MJ, McKenzie JE, Bossuyt PM, Boutron I, Hoffmann TC, Mulrow CD (2021). The PRISMA 2020 statement: an updated guideline for reporting systematic reviews. Int J Surg.

[42] Falagas ME, Pitsouni EI, Malietzis GA, Pappas G (2008). Comparison of PubMed, Scopus, web of Science, and Google Scholar: strengths and weaknesses. FASEB J.

[43] Grégoire G, Derderian F, Le Lorier J (1995). Selecting the language of the publications included in a meta-analysis: is there a tower of Babel bias?. J Clin Epidemiol.

[44] Arias MM (2018). Aspectos metodológicos Del metaanálisis (1). Pediatr Aten Primaria.

[45] Covidence. Covidence systematic review software. Veritas Health Innovation, Australia. 2014. https://www.covidence.org/. Accessed 28 Feb 2022.

[46] Selan D, Jakobsson U, Condelius A (2017). The Swedish P-CAT: modification and exploration of psychometric properties of two different versions. Scand J Caring Sci.

[47] Beaton DE, Bombardier C, Guillemin F, Ferraz MB (2000). Guidelines for the process of cross-cultural adaptation of self-report measures. Spine (Phila Pa 1976).

[48] Guillemin F (1995). Cross-cultural adaptation and validation of health status measures. Scand J Rheumatol.

[49] Hambleton R, Merenda P, Spielberger C (2005). Adapting educational and psychological tests for cross-cultural assessment.

[50] Muñiz J, Elosua P, Hambleton RK (2013). International test commission guidelines for test translation and adaptation: second edition. Psicothema.

[51] Rosengren K, Brannefors P, Carlstrom E (2021). Adoption of the concept of person-centred care into discourse in Europe: a systematic literature review. J Health Organ Manag.

[52] Alharbi T, Olsson LE, Ekman I, Carlström E (2014). The impact of organizational culture on the outcome of hospital care: after the implementation of person-centred care. Scand J Public Health.

[53] Bensbih S, Souadka A, Diez AG, Bouksour O (2020). Patient centered care: focus on low and middle income countries and proposition of new conceptual model. J Med Surg Res.

[54] Stranz A, Sörensdotter R (2016). Interpretations of person-centered dementia care: same rhetoric, different practices? A comparative study of nursing homes in England and Sweden. J Aging Stud.

[55] Zhou LM, Xu RH, Xu YH, Chang JH, Wang D (2021). Inpatients’ perception of patient-centered care in Guangdong province, China: a cross-sectional study. Inquiry.

[56] Marsh HW, Morin AJ, Parker PD, Kaur G (2014). Exploratory structural equation modeling: an integration of the best features of exploratory and confirmatory factor analysis. Annu Rev Clin Psychol.

[57] Asparouhov T, Muthén B (2009). Exploratory structural equation modeling. Struct Equ Model Multidiscip J.

[58] Cabedo-Peris J, Martí-Vilar M, Merino-Soto C, Ortiz-Morán M (2022). Basic empathy scale: a systematic review and reliability generalization meta-analysis. Healthc (Basel).

[59] Flora DB (2020). Your coefficient alpha is probably wrong, but which coefficient omega is right? A tutorial on using R to obtain better reliability estimates. Adv Methods Pract Psychol Sci.

[60] McNeish D (2018). Thanks coefficient alpha, we’ll take it from here. Psychol Methods.

[61] Hayes AF, Coutts JJ (2020). Use omega rather than Cronbach’s alpha for estimating reliability. But… Commun Methods Meas.

[62] Cronbach LJ (1951). Coefficient alpha and the internal structure of tests. Psychometrika.

[63] McDonald R (1999). Test theory: a unified approach.

[64] Polit DF (2014). Getting serious about test-retest reliability: a critique of retest research and some recommendations. Qual Life Res.

[65] Ceylan D, Çizel B, Karakaş H (2020). Testing destination image scale invariance for intergroup comparison. Tour Anal.

[66] Rönkkö M, Cho E (2022). An updated guideline for assessing discriminant validity. Organ Res Methods.

[67] Hubley A, Zumbo B, Zumbo B, Hubley A (2017). Response processes in the context of validity: setting the stage. Understanding and investigating response processes in validation research.

[68] Messick S, Philips G (1996). Validity of performance assessments. Technical issues in large-scale performance assessment.

[69] Moss PA (1998). The role of consequences in validity theory. Educ Meas Issues Pract.

[70] Cronbach L, Wainer H (1988). Five perspectives on validity argument. Test validity.

[71] Birkle C, Pendlebury DA, Schnell J, Adams J (2020). Web of Science as a data source for research on scientific and scholarly activity. Quant Sci Stud.

[72] Bramer WM, Rethlefsen ML, Kleijnen J, Franco OH (2017). Optimal database combinations for literature searches in systematic reviews: a prospective exploratory study. Syst Rev.

[73] Web of Science Group. Editorial selection process. Clarivate. 2024. https://clarivate.com/webofsciencegroup/solutions/%20editorial-selection-process/. Accessed 12 Sept 2022.

